# Dementia screening protocol for primary care in South America: a Delphi consensus study

**DOI:** 10.3389/fpubh.2026.1798111

**Published:** 2026-04-08

**Authors:** Belen Custodio, Rosa Montesinos, Paulo Caramelli, Ismael Calandri, Sonia Maria Dozzi Brucki, Claudia Kimie Suemoto, Ricardo Allegri, Andrea Slachevsky, José F. Parodi, Patricia Morsch, Clarissa Willets, María Sofía Cuba Fuentes, Nilton Custodio

**Affiliations:** 1 Research Department, Instituto Peruano de Neurociencias, Lima, Peru; 2 Research Department, Equilibria, Lima, Peru; 3 Centro de Investigación del Envejecimiento, Facultad de Medicina, Universidad de San Martín de Porres, Lima, Peru; 4 Behavioral and Cognitive Neurology Unit, Faculdade de Medicina, Universidade Federal de Minas Gerais, Belo Horizonte, Brazil; 5 Servicio de Neurología Cognitiva, Instituto Neurológico Fleni, Buenos Aires, Argentina; 6 Cognitive and Behavioral Neurology Unit, Hospital das Clínicas, Faculdade de Medicina, Universidade de São Paulo, São Paulo, Brazil; 7 Division of Geriatrics, Faculdade de Medicina, Universidade de São Paulo, São Paulo, Brazil; 8 Departamento de Neurociencias, Universidad de la Costa (CUC), Barranquilla, Colombia; 9 Gerosciences Center for Brain Health and Metabolism (GERO), Santiago, Chile; 10 Memory and Neuropsychiatric Center, Neurology Department, Del Salvador Hospital, School of Medicine, Universidad de Chile, Santiago, Chile; 11 Neuropsychology and Clinical Neuroscience Laboratory (LANNEC), Interdisciplinary Nucleus for Physiology, Biophysics, and Pathophysiology – ICBM, Neuroscience and East Neuroscience Departments, Faculty of Medicine, Universidad de Chile, Santiago, Chile; 12 Neurology and Psychiatry Department, Clínica Alemana-Universidad del Desarrollo, Santiago, Chile; 13 Life Course Unit, Health Systems and Services Department, Pan American Health Organization, Washington, DC, United States; 14 Family Medicine Residency Program, Faculdade de Medicina, Universidade de São Paulo, São Paulo, Brazil; 15 Center for Research in Primary Health Care, Universidad Peruana Cayetano Heredia, Lima, Peru

**Keywords:** dementia, Latin America, modified-Delphi technique, primary care, screening

## Abstract

**Introduction:**

Rapid population aging in South America (SA) highlights the need for effective dementia detection strategies. Primary care is essential for early identification, yet no shared, regionally contextualized approach exists. This study aimed to develop expert-based recommendations for dementia screening in SA primary care.

**Methods:**

A two-round modified Delphi process involved 20 SA experts from different specialties. Round 1 explored open-ended and ranking questions on cognitive and functional screening tools, cut-offs, referral criteria, administration time, and professional roles. Round 2 sought consensus (≥75%) on items informed by Round 1.

**Results:**

Consensus was reached on using an initial screening question to identify potential cognitive decline, the duration of a brief screening battery, and the primary care team’s roles. Experts emphasized the need for educational, cultural, and sensory adaptations and proposed strategies for patients without reliable informants. An agreement on functional assessment tools and some cut-off points was not achieved.

**Discussion:**

These recommendations provide a structured, flexible, and culturally sensitive framework for primary care dementia screening, supporting timely detection, timely referral and integration into person-centered approaches to healthy aging, while highlighting areas requiring further research and standardization.

## 1 Introduction

Dementia is a growing public health concern around the world, disproportionately affecting low- and middle-income countries (LMICs). Latin America (LA), which comprises South America, Central America, Mexico, and the Caribbean, is projected to have over 27 million people living with dementia by 2050 (1). Recent studies suggest that the prevalence of dementia among adults aged 50 and older has increased compared to earlier estimates, reflecting both population growth and aging, as well as a high burden of risk factors such as low educational attainment, cardiovascular conditions, and social inequalities (2, 3). Given that the likelihood of developing dementia rises sharply after the age of 65, the rapid demographic transition in LA countries is expected to dramatically increase the number of affected individuals in the coming decades, which will make dementia a leading cause of disability among older adults, imposing significant challenges on healthcare systems, families, and societies at large (1, 4). This scenario highlights the urgent need for effective strategies to detect and manage dementia.

Timely detection has been proven to be highly beneficial, enabling timely access to pharmacological and non-pharmacological treatments, facilitating planning for future care, and improving quality of life for patients and their caregivers, while also mitigating broader social and economic impacts (5). However, dementia is frequently underdiagnosed or diagnosed late (6). Globally, it is estimated that 75% of people living with dementia remain undiagnosed (7). In Brazil, for instance, over 70% of older adults with dementia are not formally diagnosed (8). This is primarily driven by limited awareness, stigma surrounding aging and cognitive impairment, and insufficient training among healthcare professionals, particularly in primary care settings (7, 9, 10).

The diagnosis of dementia syndrome is clinical, based on clinical history and cognitive and functional assessments, with details provided by a close informant. Brief cognitive and functional screening tools (BCS and BFS) can substantially improve the accuracy and timeliness of diagnoses, especially when conducted in primary care (5). To date, a wide range of BCS and BFS are available, each evaluating different aspects of cognition and daily functioning (11). However, many of these tools were developed and validated outside of South America (SA), and evidence on their performance in local contexts, especially in rural areas or among individuals with low educational levels, where cultural and linguistic differences, literacy demands, and unfamiliar task formats may affect test accuracy, is limited (12). As a result, clinicians across the region often rely on a heterogeneous mix of tools, highlighting the need for careful consideration when selecting screening tools for diverse primary care populations.

Although several initiatives have emerged across the region, most South American countries still lack an implemented national dementia plan (13). An exception is Chile, which enacted its National Dementia Plan in 2017 with the aim of providing continuous care across all levels of the health system (14). Beyond the absence of formal plans, health disparities and social determinants of health, including low educational attainment, cardiometabolic conditions, and social isolation, disproportionately influence cognitive aging and functional ability in SA populations, often more strongly than traditional risk factors such as age and sex (15, 16). This heterogeneity in risk factors is particularly pronounced across the region, underscoring the need for context-sensitive approaches to dementia detection. Existing international guidelines (17, 18) recommend screening tools that were largely developed and validated in populations with higher educational attainment, and are therefore heavily influenced by literacy and formal education, limiting their applicability in SA populations where low educational attainment remains prevalent (12). As a result, despite the increasing recognition of dementia as a public health priority in the region, there is currently no standardized, region-wide protocol for dementia screening in primary care that accounts for these contextual differences. The recent advances in diagnostic criteria for Alzheimer’s disease (AD), including the use of biomarkers (19, 20), as well as the emergence of novel disease-modifying treatments, further underscore the need for coherent protocols.

In a regional context where a growing number of people are living with dementia alongside rapid aging and social inequality; primary care still lacks a shared, contextualized pathway for early detection. Therefore, this study aimed to develop expert-based recommendations for a brief and feasible dementia screening protocol tailored to primary care settings in South America (SA). Using a Delphi approach with clinicians and researchers from across the region, we sought to identify the cognitive and functional tools considered most appropriate for use in low- and medium-resource environments that could support earlier and more reliable detection of dementia in diverse SA populations.

## 2 Materials and methods

### 2.1 Study design

This study employed a modified Delphi methodology to reach expert consensus on a screening protocol for the detection of dementia in primary care settings in SA. The Delphi technique is a structured, iterative process that gathers opinions from experts through multiple rounds of anonymous questionnaires (21, 22). This method is widely used in health research to develop guidelines, standards, or protocols in areas where empirical evidence is limited or heterogeneous (21, 22).

### 2.2 Generation of an initial instrument to obtain consensus

The core team consisted of 12 health professionals, including neurologists, geriatricians, primary care physicians, and public health professionals from four South American countries (Peru, Brazil, Argentina and Chile). This group developed the initial questionnaire drawing on: (1) a review of international dementia diagnostic and screening guidelines; (2) expertise in cognitive and functional assessment tools widely used in the region; and (3) contextual considerations relevant to South American primary care systems, including variations in educational attainment, time constraints during consultations, and limited availability of diagnostic resources. The first version of the instrument was pilot-tested within the core team to ensure clarity and relevance. This pilot phase focused exclusively on refining wording and structure, without influencing the content that would later be evaluated independently by the expert panel. The final questionnaire covered all components necessary for developing a feasible and context-appropriate dementia screening protocol, which are summarized in Table 1.

**Table 1:** Topics covered on the initial instrument developed by the core team.

#	Topic
1.	Identification of the target population
2.	Agreement with an introductory screening question
3.	Ranking of the local validated BCS and BFS Tools including: Mini Mental State Examination (MMSE) (51) Montreal Cognitive Assessment (MoCA) (52) Brief Cognitive Screening Battery (BCSB-Brazil) (53) Consortium to Establish a Registry for Alzheimer’s Disease (CERAD) (54) Informant Questionnaire on Cognitive Decline (IQCODE) (55) Clock drawing test (CDT) (56) Ascertain Dementia 8 (AD8) (57) Rowland Universal Dementia Assessment Scale (RUDAS) (58) 10-point cognitive screener (10-CS) (59)
4.	Proposed cut-offs and educational/language adjustments
5.	Selection of subtest for a minimal battery
6.	Referral criteria
7.	Administration time and team roles
8.	Preferred tools by population subgroup
9.	Procedures for patients without a reliable informant
10.	Additional contextual or ethical considerations

### 2.3 Selection of the expert panel

Panel members were selected through a purposive sampling guided by the core team. A total of 20 healthcare professionals were invited via email to participate. Eligible experts met the following criteria: (1) a minimum of 5 years of experience in the field of dementia, (2) specialty training in Neurology, Psychiatry, Geriatrics, or Family Medicine (3) current clinical and/or academic practice in a South American country. Participation was voluntary and anonymous. Experts who completed Round 1 were automatically invited to Round 2.

### 2.4 Delphi process

Consensus was defined *a priori* as ≥75% convergence in responses, regardless of whether participants agreed or disagreed with the statement. Questions that reached the threshold in Round 1 were considered to have achieved preliminary consensus.

The questionnaires were administered using Typeform©,^1^ an online survey platform, with an estimated completion time of 15 min per round. Each Delphi round followed a predefined timeline, allocating 15 days for panelists to submit their responses, followed by approximately 1 week for the research team to review comments, synthesize feedback, and construct the questionnaire for the subsequent round.

The first round consisted of a questionnaire developed by the core team and was designed as an exploratory phase to gather a wide range of opinions and identify key themes for the screening protocol. Most questions in this round were open-ended, allowing participants to freely suggest components of a minimum screening protocol, including cognitive and functional assessments, cut-off points for specific subgroups, and contextual considerations relevant to low-resource primary care settings. Responses from this exploratory round were then synthesized by the core team to generate structured questions and options for Round 2, aimed at achieving consensus.

Round 2 was designed to refine and validate the results from Round 1. Participants received a summary of the aggregated group responses from the previous round. They were then asked to evaluate their agreement with the items proposed in Round 1. Given the heterogeneity across countries in terms of resources, population characteristics, and clinical practices, for the questions about the selection of the most relevant BCS and BFS, panelists were asked to select the three most relevant options for their context to capture broader expert preferences while acknowledging regional variability. In all cases, the consensus threshold was consistently applied. This round aimed to achieve convergence among panel members and to finalize the recommended components of the screening protocol. Items that did not reach the consensus threshold were reported as areas of disagreement. The questionnaires for Rounds 1 and 2 are available in the Supplementary material.

### 2.5 Statistical analysis

Quantitative data from Likert-scale, ranking, and Yes/No responses were analyzed descriptively using frequencies and percentages to determine the level of agreement for each item. For the Likert-scale questions, the response options “agree” (4) and “strongly agree” (5) were combined into a single “agree” category for analysis, in line with the consensus threshold definition. Qualitative responses to open-ended questions were analyzed using a rapid content analysis approach (23) to identify common themes that informed the development of items for Round 2. All descriptive analyses were performed using Microsoft Excel.

### 2.6 Ethics statement

All experts provided consent to share their expertise as part of the Delphi process. This manuscript involves expert opinion rather than research on human subjects; therefore, ethical approval was not obtained.

## 3 Results

Twenty experts from 10 countries of SA (Argentina, Bolivia, Brazil, Chile, Colombia, Ecuador, Paraguay, Peru, Uruguay, and Venezuela), two per country, were invited to participate. All the invited completed Round 1, and 19 completed Round 2. Participants included neurologists (55%), geriatricians (30%), psychiatrists (10%), and family medicine physicians (5%). Regarding clinical experience in dementia care, four experts reported 5–10 years of experience, while the remaining 16 had more than 10 years.

### 3.1 Round 1 results

**Figure 1:**
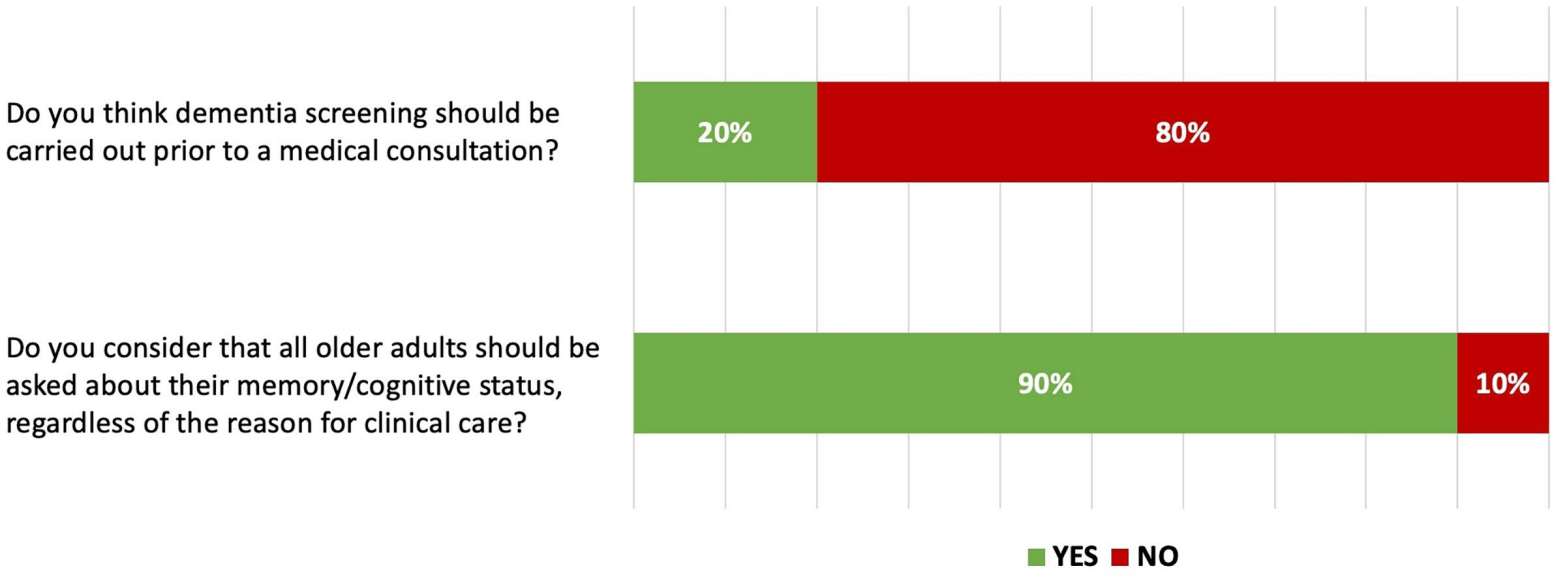
Yes/no questions that reached consensus in Round 1.

In Round 1, the panel responded to 18 questions: three yes/no items, two ranking questions for prioritizing cognitive and functional tests, and 13 open-ended questions. The results from the ranking and open-ended questions informed the development of the Round 2 questionnaire. Of the three yes/no questions, two reached consensus (Figure 1).

The ranking questions identified the top five cognitive tests as: Montreal Cognitive Assessment (MoCA), Mini-Mental State Examination (MMSE), Clock Drawing Test (CDT), Brief Cognitive Screening Battery (BCSB–Brazil), and the Rowland Universal Dementia Assessment Scale (RUDAS). Among functional assessments, the highest-ranked instrument was the Pfeffer Functional Activities Questionnaire (PFAQ), with an additional scale proposed under “Other” being Lawton and Brody. All these tools were considered when formulating the structured questions for Round 2.

The next 13 open-ended questions yielded diverse insights. For the item on establishing cut-off points by educational level, responses were highly heterogeneous with several omissions. Based on this, the research team decided that Round 2 would present established cut-offs for each instrument so that experts could indicate their level of agreement. Concerning factors requiring specific adjustments, educational level was mentioned by 85% of panelists; all other factors identified were carried forward to the next round.

For the questions asking participants to (1) define a minimum brief battery, (2) identify clinical signs that should prompt referral to specialized care, (3) suggest an acceptable maximum duration for the brief battery, (4) specify who should administer the screening question and cognitive tests, and (5) propose an approach for individuals without an available informant, the most frequently mentioned responses were extracted and incorporated into Round 2.

When addressing specific populations, the most highly ranked strategies were selected for rural and low-education populations. Regarding Indigenous groups or individuals whose primary language is not Spanish or Portuguese, 70% of panelists indicated that currently available tools are not adequately adapted to these languages. Therefore, the related Round 2 question was reformulated to focus on appropriate interim strategies while culturally and linguistically adapted tools are developed. For highly educated populations, 60% of panelists identified the MoCA as the most suitable tool for detecting dementia, and it was included in the next round. Finally, responses concerning additional ethical, cultural, or linguistic considerations were synthesized and incorporated into the corresponding Round 2 items.

### 3.2 Round 2 results

**Figure 2:**
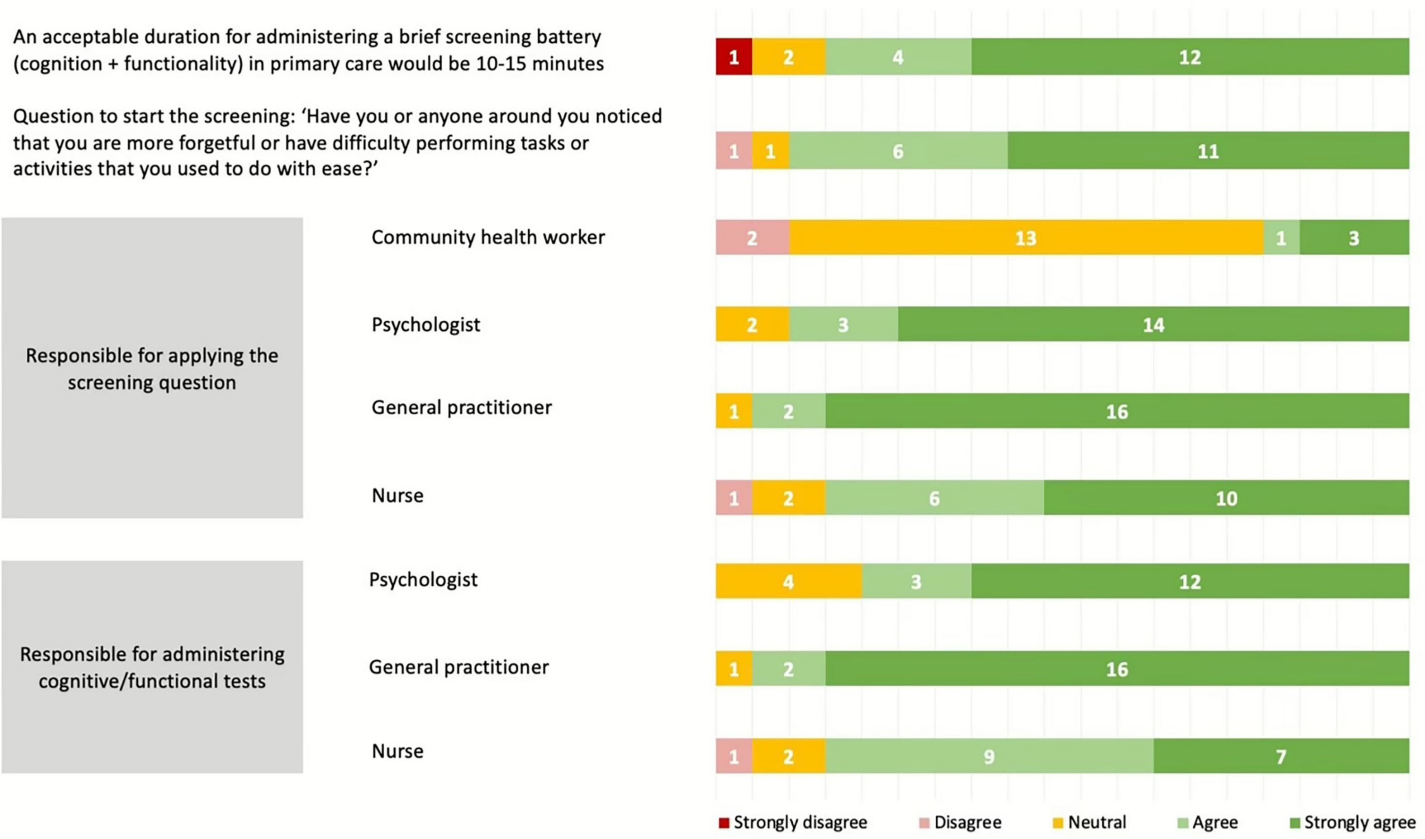
The responsibiles for administering the screening question and tests.

Several key components of the dementia screening protocol achieved consensus in this round. Eighty-five percent of panelists agreed with the proposed question to start the screening process: “Have you or anyone around you noticed that you are more forgetful or have difficulty performing tasks or activities that you used to do with ease?”. Similarly, 80% agreed that a brief screening battery, encompassing both cognitive and functional assessments, could be administered within 10–15 min in primary care settings. The responsibilities for administering the screening question and tests were clearly defined, with general practitioners (GPs), psychologists, and nursing staff identified as the main personnel, while community health workers were not recommended by the majority of experts. Detailed results for this section are presented in Figure 2.

Regarding cognitive assessment tools, the three tests identified as most relevant for primary care were the MoCA, MMSE, and CDT. However, consensus was not reached regarding the preferred tool for functional assessment, nor on defining a minimal brief battery combining both cognitive and functional evaluations.

Concerning cutoff points according to educational level and referral criteria to specialized care, mixed results were observed, with some items reaching consensus and others not. These results are detailed in Table 2. Panelists reached consensus that context, language, and sensory limitations are key factors to consider when adapting the protocol to local settings. They also agreed on the need for the protocol to be brief, simple, flexible, culturally sensitive, include clear referral pathways, and provide specific training for primary care teams.

**Table 2:** Expert panel responses on education-specific cut-off points and referral criteria to specialized care.

Brief cognitive screening tool	Cut-off	Agreed	Disagreed	Consensus
Cut-off points according educational level				
RUDAS*	For people with 6 or more years of education, RUDAS < 21	16	3	YES
	For people with less than 6 years of education, RUDAS < 18	15	3	YES
MoCA†	For people with 6 or more years of education, MoCA < 20	15	3	YES
	For people with less than 6 years of education, MoCA < 15	12	7	NO
MMSE‡	For people with 6 or more years of education, MMSE < 19	12	7	NO
	For people with less than 6 years of education, MMSE < 14	8	11	NO
Criteria for referral to specialized care				
Behavioral symptoms		15	4	YES
Rapid progression (less than 3 months)		17	2	YES
Motor disorder		16	3	YES
Being under 60 years of age		16	3	YES
Diagnostic uncertainty		19	0	YES
Language disorder		14	5	NO
Polypharmacy and/or multiple associated comorbidities		8	11	NO
Any patient confirmed with dementia who does not respond to first-line treatment after 6 months		11	8	NO

*Rowland Universal Dementia Assessment Scale; †Montreal Cognitive Assessment; ‡Mini-Mental State Examination.

For preferred tests in specific populations, the only item reaching consensus referred to highly educated populations, where 85% of panelists considered the MoCA the most appropriate cognitive assessment. For rural populations and individuals with low literacy or illiteracy, panelists were asked to select their top three options; however, none reached the consensus threshold. Similarly, for strategies to use until tools are adapted and validated for Indigenous populations or patients whose primary language is not Spanish or Portuguese, none of the proposed alternatives achieved 75% agreement.

Lastly, regarding procedures for patients without a reliable informant, panelists reached consensus on several strategies, including identifying a valid informant via a social worker, conducting telephone interviews with family members, using clinical questions to collect cognitive and functional information, and administering screening tests.

## 4 Discussion

This Delphi study aimed to develop a consensus-based framework for dementia screening in primary care across SA. Panelists reached consensus on how to start the screening process, the optimal administration time, and the roles of primary care professionals, as well as strategies to ensure cultural and linguistic sensitivity and to guide assessment when no reliable informant is available. Other areas, such as preferred functional assessment tools, educationally adjusted cutoff points, referral criteria, and test selection for specific populations, showed more variable agreement, reflecting the heterogeneity of clinical practice across the region.

In any Delphi procedure, the choice of the panel members is critical and a potential source of bias; that is, results may be skewed toward a particular outcome related to the panelists’ primary area of expertise (24, 25). To prevent this, we chose *a priori* the medical specialties dealing with dementia’s clinical diagnosis based on BCS and BFS. This balanced approach reduced the weight of idiosyncratic votes, and allowed a sufficient number of participants to guarantee stable results (26–28). As an intrinsic limitation, each panelist may be an expert of the pertinent BCS tool or BFS tool of interest and little of the others.

The panel recognized the importance of the initial screening question (*Have you, or anyone around you, noticed that you are more forgetful or have difficulty performing tasks or activities that you used to do with ease?*) and identified GPs (90%), psychologists (85%), and nurses (80%) as primary care team members to perform this question and apply the BCS and BFS tools. In this sense, a reliable screening for dementia, delivered by primary care professionals, rather than by dementia specialists, as recommended by the WHO (29, 30) and adaptable across multiple contexts, could reduce barriers to timely diagnosis and enhance care planning. In Peru, the ‘IMPACT Salud’ study will be the first in SA to evaluate the validation and cost-effectiveness of a community-based mHealth-enabled tool for cognitive and functional impairment detection delivered by CHWs, including AD8, RUDAS, and PFAQ (31). Additionally, panelists endorsed the notion that a time constraint is often an issue for GPs, nurses, or psychologists as it relates to diagnosing dementia, and they agree with 10 to 15 min to administer a BCS and/or BFS tool in primary care. The time allocated for a typical office visit makes it challenging to perform a cognitive assessment and diverse studies are intended to assist GPs in creating simple strategies (32, 33) to manage cognitive complaints and to determine when to refer patients to a specialist consultation (34).

Furthermore, the panelists agreed that BCS and BFS tools should be adapted according to educational level, local language, and sensory limitations. This implies that harmonization requires culturally validated tools not influenced by education or literacy (35). Tools that minimize the effect of education, literacy, and culture have been developed worldwide (36–38), and in SA (12, 39). However, the literature indicates that more research is required to validate these tools and determine their suitability across a wide range of settings. This panel considered three BCS and two BFS tools as most appropriate for dementia screening in primary care in their context: MMSE (90%), MoCA (90%), and CDT (70%), but only 60% recommend PFAQ or Lawton IADL as a BFS tool.

The MMSE is one of the most frequently used tools for screening for dementia for several reasons. However, its scores are heavily influenced by formal schooling and do not include executive function domains, so it may not accurately detect cases of mild cognitive impairment (MCI) (12). The panel reached consensus on MoCA cut-off scores for individuals with more than 6 years of education and on RUDAS for individuals with high and low education levels, including illiterate individuals; however, no consensus was reached on the MMSE. In Brazil, ROC analyses revealed that the MoCA can accurately distinguish controls from individuals with dementia, with high sensitivity (90%) and specificity (77%) considering a very low cutoff score (= 15). Accuracy was higher among participants with more than 4 years of education, reaching 100% sensitivity, indicating that MoCA performance may be influenced by educational level. In the Chilean population, normative data for the MoCA and proposal of cut-off points for different age ranges to discriminate normal cognitive performance from neurocognitive disorders and adjusted for education level, would assist in the use of the test and reduce the rate of false positives (40). The MoCA adaptation process assessment revealed limited reporting on the translation steps involved, with few studies detailing the original author’s involvement, professional translation, pilot testing, or healthcare professional input. A systematic review suggests the use of suitable cutoff scores, educational level-based scoring adjustments, and cultural awareness (41). One tool that stood out as potentially promising was the RUDAS, demonstrating that literate and illiterate participants performed similarly in this tool. The RUDAS was developed for use in culturally and linguistically diverse populations. It has been validated in more than 20 countries, in at least 16 languages, and has been validated in SA, including Brazil (42), Peru (43, 44), and Chile (45).

In SA, varying MMSE cut-off points for low-educated individuals likely explain the lack of consensus on its use. In a low-educated Peruvian cohort, a score of 19 optimally distinguished controls from MCI (sensitivity 87.5%, but 35% false positives), while a score of 14 differentiated MCI from dementia (sensitivity 100%, specificity 98.4%) (44). In Chile, without specifying the size of the population with low education (comparing 1–12 years of education with more than 13 years), a higher MMSE score was reported in subjects with 13 or more years of schooling, with a difference of about 3 points in the tenth percentile (46). Future research should focus on determining the sensitivity and specificity of the tools in terms of differentiating between cognitive unimpaired aging, MCI and various types of dementia, the effect of translation, and the impact on confounding variables.

We note the panel’s lack of consensus in suggesting a BFS tool given the importance of caregiver-informed functional assessments as part of dementia evaluations. Informant-reported functional changes can sometimes even outpace measurable cognitive decline, indicating that tools like the PFAQ capture meaningful decline that BCS tools might underestimate. This is especially relevant given that functional impairment is a core criterion distinguishing dementia from prodromal stages (47). In SA, PFAQ composed of 11 items assessing instrumental activities of daily living was validated in Chile (48), and has been used in the ReDLat study (49). Recently, a study characterized functional capacity across the continuum from normal aging to MCI, and two subtypes of dementia: Alzheimer’s disease (AD) and frontotemporal dementia (FTD) in a Peruvian cohort, with a particular focus on distinguishing the profiles of AD and FTD (50).

These results establish the foundational assumptions for the diagnostic workflow. Based on the iterative Delphi process and updated clinical evidence, this framework aims to support primary healthcare professionals using the BCS and BFS tools to classify individuals as cognitively healthy, with MCI, or with dementia, and to incorporate these outcomes into subsequent etiological diagnosis and non-pharmacological management. If adopted in routine care, this consensus-based pathway could shift dementia case-finding from a fragmented practice to a structured, person-centered process, reducing diagnostic delays and reactive care. Moreover, it positions dementia screening within a broader healthy aging and Integrated Care for Older People (ICOPE) approach that emphasizes early identification of cognitive and functional decline to protect intrinsic capacity, support aging in place, and and align decisions with what matters most to older adults (30).

### 4.1 Limitations

Although the Delphi procedure is widely used to establish clinical recommendations, it is not devoid of limitations. Response bias can be related to the mix of panel expertise, as mentioned above, and to the implicitly suggestive wording of the questions. The mitigation measures we took consisted of piloting the first-round questionnaire, as well as inviting a diverse panel of experts from all disciplines involved in clinical decision-making. Despite representation from multiple countries, most panelists were based in the country’s capital and affiliated with specialized centers. This may introduce a geographical bias, as participants’ preferences could be shaped by the resources and tools typically available in their settings. For instance, the selection of cognitive and functional screening instruments may have been influenced not only by their psychometric properties but also by local availability, familiarity, or institutional practice patterns. A more geographically and institutionally diverse panel may provide a greater range of responses and experiences upon which to draw. Another limitation was the predominance of neurologists, with only 5% of the panel comprising primary care physicians. Although specialists have extensive expertise in dementia diagnosis and management, they may have limited familiarity with the specific constraints, workflows, and resources of primary care settings across SA. In addition, it is important to consider the heterogeneous role of GPs in primary care across SA countries, as in many contexts they may serve as the first point of contact for patients with cognitive impairment, while in others the assessment is primarily performed by specialists. Including a broader range of health professionals involved in dementia care, as well as people with lived experience of dementia, could help capture diverse perspectives and make the recommendations more applicable across different care contexts.

### 4.2 Conclusion

This Delphi study provides the first South American–level consensus on dementia screening in primary care, offering a structured, flexible, and culturally sensitive framework. Experts agreed on the use of an initial screening question, a brief cognitive assessment battery, clear roles for general practitioners, psychologists, and nurses, as well as strategies for patients without a reliable informant. Although consensus was not reached on functional assessment tools and some cut-off points, the recommendations offer a foundation for standardized screening, supporting early detection and timely referral. Implementation of this protocol could enhance diagnostic accuracy, reduce delays, and integrate dementia screening into broader healthy aging and person-centered care initiatives, while providing a reference framework for healthcare professionals and policy makers across diverse South American contexts.

## Data Availability

The raw data supporting the conclusions of this article will be made available by the authors, without undue reservation.

## Footnotes

1
https://www.typeform.com
