# Supplementary material for: Dementia screening protocol for primary care in South America: a Delphi consensus study
Source: Front Public Health. 2026 Apr 8;14:1798111. doi: 10.3389/fpubh.2026.1798111 (PMC13099327; doi:10.3389/fpubh.2026.1798111)
Supplement: Supplementary file 1 [file Table_1.docx]

Supplementary Material

# Round 1 Questionnaire

## Identification of the target population

Do you believe that all older adults should be asked about their memory/cognitive status, regardless of the reason for clinical care?

- YES
- NO

Do you think dementia screening should be carried out prior to a medical consultation?

- YES, it should be done before entering the doctor's office.

**IF YES:**

Where should it take place? ___________________

- NO, it must be done during the medical consultation.

Do you think dementia screening should begin with clinical questions during the medical consultation?

- YES

**IF YES:**

Do you agree with this question? 🡪 “Has a family member, friend, or yourself noticed that you are more forgetful and additionally have difficulty doing tasks or activities that you used to do with ease?”

- Yes
- No

**IF NO:** If you disagree with the proposed question, what would you suggest?

- NO

## Brief cognitive and/or brief functional screening tools to consider

From the following list, please rank the cognitive tests in order of relevance for dementia screening in primary care in your context.

- - Mini Mental State Examination – MMSE
  - Montreal Cognitive Assessment – MoCA
  - Brief Cognitive Screening Battery - BCSB (Brazil)
  - Consortium to Establish a Registry for Alzheimer’s Disease – CERAD
  - Informant Questionnaire on Cognitive Decline – IQCODE
  - Clock drawing test
  - Ascertain Dementia 8 - AD8
  - Rowland Universal Dementia Assessment Scale – RUDAS
  - 10-point cognitive screener - 10-CS
  - Other: __________________

From the following list, please rank up the functional tests in order of relevance for dementia screening in primary care in your context.

- - Pfeffer Functional Activities Questionnaire PFAQ
  - Ascertain Dementia 8 - AD8
  - Informant Questionnaire on Cognitive Decline – IQCODE
  - Other: __________________

## Cut-off thresholds

For each test mentioned in the previous questions, if you deem it necessary, suggest cut-offs for suspected dementia, and specify whether they should be adjusted for education level or language (e.g., “MMSE < 20 for ≤6 years of schooling”).

## Adjustments for education/language

In your experience, which factors (educational level, language, rurality, culture) require specific adjustments, and how would you implement them in practice? Describe up to 3 practical recommendations.

## Minimum combination of subtests

If you had to define a minimal brief battery (≤15 minutes) for probable diagnosis in low-resource settings, which subtests would you include (e.g., 3-word recall, animal verbal fluency, clock drawing) and why?

## Referral criteria

What clinical signs, test results, or other factors would lead you to refer a patient with cognitive complaints to specialist care (e.g., neurology, geriatrics, psychiatry)? Please list the main reasons you would consider.

What findings or situations would lead you to request (or refer for) a full evaluation, such as neuropsychological testing or neuroimaging? Please describe the key criteria you usually consider.

## Maximum acceptable time and team resources

In your opinion, what is the maximum acceptable duration for administering a brief cognitive/functional screening battery in primary care? Please indicate the approximate time (in minutes) and explain why you consider this timeframe acceptable in your practice (e.g., workload, patient tolerance, consultation length, health system constraints).

In your opinion, which primary care team members could ask the screening questions?

In your opinion, which primary care team members could perform cognitive tests?

## Preferred tests by context

In your health system context, are there specific cognitive or functional screening tools that you consider particularly appropriate for different populations? In particular, please comment on:

- - Rural populations and individuals with low education or illiteracy
  - Indigenous populations or patients whose primary language is not Spanish
  - Highly educated populations

## Other recommendations

Are there any ethical, cultural, or logistical aspects we should consider when designing the protocol? (free comment).

Recommend a procedure for individuals without a reliable proxy

# Round 2 Questionnaire

## Start of Screening

Based on the results of the first round, all older adults should be asked about their memory or cognitive status, regardless of the reason for the visit. Dementia screening should be conducted during the medical consultation, starting with a clinical question. With this in mind, please indicate your level of agreement with the following question to start the screening:

- *“Have you or anyone around you noticed that you are more forgetful or have difficulty performing tasks or activities that you used to do with ease?”*

Where 1 represents the lowest level of agreement and 5 the highest level.

| 1 | 2 | 3 | 4 | 5 |
| --- | --- | --- | --- | --- |

## Priority Cognitive and Functional Tests

From the results of the first round, the cognitive tests most frequently ranked at the top were: MoCA, MMSE, Clock Drawing Test, BCSB, and RUDAS.

Please select the three tests most relevant to your primary care context (max. 3):

- Montreal Cognitive Assessment – MoCA
- Mini Mental State Examination – MMSE
- Clock Drawing Test
- Brief Cognitive Screening Battery – BCSB-Brazil
- Rowland Universal Dementia Assessment Scale – RUDAS

In the first round, the "Pfeffer Functional Activities Questionnaire – PFAQ" was the most mentioned for assessing functionality in primary care.

Please indicate your level of agreement with the following statement:

- *“Pfeffer Functional Activities Questionnaire – PFAQ is an appropriate tool to assess functionality in the dementia screening protocol in primary care."*

Where 1 represents the lowest level of agreement and 5 the highest level.

| 1 | 2 | 3 | 4 | 5 |
| --- | --- | --- | --- | --- |

Another test mentioned during the first round was “Lawton & Brody.”

Please indicate your level of agreement with the following statement:

- *"Lawton & Brody is an appropriate tool to assess functionality in the dementia screening protocol in primary care."*

Where 1 represents the lowest level of agreement and 5 the highest level.

| 1 | 2 | 3 | 4 | 5 |
| --- | --- | --- | --- | --- |

## Cut-off Points Adjusted by Educational Level

The cut-off point for the Montreal Cognitive Assessment – MoCA, discriminating cognitively healthy controls from dementia based on education level, is:

|  | | **YES** | **NO** |
| --- | --- | --- | --- |
| **MoCA** | For individuals with 6 or more years of education, MoCA < 20 |  |  |
|  | For individuals with less than 6 years of education, MoCA < 15 |  |  |

The cut-off point for the Mini Mental State Examination – MMSE, discriminating cognitively healthy controls from dementia based on education level, is:

|  | | **YES** | **NO** |
| --- | --- | --- | --- |
| **MMSE** | For individuals with 6 or more years of education, MMSE < 19 |  |  |
|  | For individuals with less than 6 years of education, MMSE < 14 |  |  |

The cut-off point for the Rowland Universal Dementia Assessment Scale – RUDAS, discriminating cognitively healthy controls from dementia based on education level, is:

|  | | **YES** | **NO** |
| --- | --- | --- | --- |
| **RUDAS** | For individuals with 6 or more years of education, RUDAS < 21 |  |  |
|  | For individuals with less than 6 years of education, RUDAS < 18 |  |  |

## Contextual Adjustments

In addition to educational level, other factors influence the dementia screening process and require specific adjustments. The most frequently mentioned were language and context. Other factors mentioned were age, sensory limitations, and sex, but less frequently.

Please rank the three most important factors to consider when adapting the protocol to your local context (max 3):

- Language
- Context (culture, rurality, occupation)
- Age
- Sensory limitations
- Sex

## Minimum Brief Battery

Based on responses about preferred cognitive and functional tests from the first round, please select the combination of 1 cognitive test and 1 functional test to define a minimum brief battery (≤15 minutes) for probable diagnosis in resource-limited settings:

**Cognitive tests:**

- Montreal Cognitive Assessment – MoCA
- Mini Mental State Examination – MMSE
- Clock Drawing Test
- Brief Cognitive Screening Battery – BCSB-Brazil
- Rowland Universal Dementia Assessment Scale – RUDAS

**Functional tests:**

- Pfeffer Functional Activities Questionnaire – PFAQ
- Ascertain Dementia 8 – AD8
- Informant Questionnaire on Cognitive Decline – IQCODE
- Lawton & Brody

## Referral Criteria

For each criterion, indicate its level of importance for referral to specialized care:

Where 1 represents the lowest level of agreement and 5 the highest level.

|  | **1** | **2** | **3** | **4** | **5** |
| --- | --- | --- | --- | --- | --- |
| Behavioral symptoms |  |  |  |  |  |
| Rapid progression (less than 3 months) |  |  |  |  |  |
| Motor disorder |  |  |  |  |  |
| Language disorder |  |  |  |  |  |
| Being younger than 60 years |  |  |  |  |  |
| Polypharmacy and/or multiple associated comorbidities |  |  |  |  |  |
| Diagnostic uncertainty of dementia |  |  |  |  |  |
| Any patient with confirmed dementia not responding to first-line treatment after 6 months |  |  |  |  |  |

## Screening Time

According to the first-round results, the most frequent option was 10–15 minutes.

Please indicate your level of agreement with the following statement:

- *"An acceptable duration to administer a brief screening battery (cognition + functionality) in primary care would be 10–15 minutes."*

Where 1 represents the lowest level of agreement and 5 the highest level.

| 1 | 2 | 3 | 4 | 5 |
| --- | --- | --- | --- | --- |

## Screening Personnel

In the first round, the primary care team members most mentioned as responsible for **asking the screening question were**: nursing staff, general practitioners, psychologists, and community health workers.

Having received prior training, indicate your level of agreement that each of the following professionals should be responsible for **asking the screening question**:

Where 1 represents the lowest level of agreement and 5 the highest level.

|  | **1** | **2** | **3** | **4** | **5** |
| --- | --- | --- | --- | --- | --- |
| Nursing staff |  |  |  |  |  |
| General practitioner |  |  |  |  |  |
| Psychologist |  |  |  |  |  |
| Community Health Worker |  |  |  |  |  |

In the first round, the primary care team members most mentioned as responsible for **administering cognitive and functional tests** were: nursing staff, general practitioners, and psychologists.

Having received prior training, indicate your level of agreement that each of the following professionals should **administer the cognitive and functional tests:**

Where 1 represents the lowest level of agreement and 5 the highest level.

|  | **1** | **2** | **3** | **4** | **5** |
| --- | --- | --- | --- | --- | --- |
| Nursing staff |  |  |  |  |  |
| General practitioner |  |  |  |  |  |
| Psychologist |  |  |  |  |  |

## Preferred Tests by Context

***Rural populations and individuals with low education or illiteracy***

In the first round, the most frequently suggested tests for rural populations or individuals with low education/illiteracy were: MMSE, RUDAS, PFAQ, Clock Drawing Test, MoCA, Five-Word Test, AD8, and Brief Cognitive Screening Battery (BCSB).

In your opinion, which of the following tests are most suitable for dementia detection in these populations? (select up to 3)

- Mini Mental State Examination – MMSE
- Rowland Universal Dementia Assessment Scale – RUDAS
- Pfeffer Functional Activities Questionnaire – PFAQ
- Montreal Cognitive Assessment – MoCA
- Five-Word Test
- Ascertain Dementia 8 – AD8
- Brief Cognitive Screening Battery – BCSB-Brazil

***Indigenous populations or patients whose primary language is not Spanish or Portuguese***

In the first round, most participants indicated that there are no validated instruments for cognitive screening in indigenous populations or speakers of languages other than Spanish or Portuguese.

Please select the strategies you consider most appropriate while the adapted tests are being developed (more than one may be selected):

- Use adapted, translated, and validated versions of existing tests when available
- Employ non-verbal or visual tasks (e.g., clock drawing, figure recognition)
- Prioritize functional assessment through informant interviews
- Include cultural mediators or translators during evaluation
- Other (please specify)

***Highly educated populations***

In the first round, the Montreal Cognitive Assessment – MoCA was identified as the most suitable test for highly educated individuals. Please indicate your level of agreement with the following statement:

- *"Montreal Cognitive Assessment – MoCA should be the cognitive test used to detect possible dementia cases in highly educated populations."*

Where 1 represents the lowest level of agreement and 5 the highest level.

| 1 | 2 | 3 | 4 | 5 |
| --- | --- | --- | --- | --- |

## Cultural, Ethical, and/or Logistical Considerations

In the first round, various aspects were identified that should be considered when implementing a dementia screening protocol in primary care, including cultural sensitivity, adaptability to diverse contexts, and logistical challenges.

Please indicate your level of agreement with each of the following statements related to protocol implementation:

Where 1 represents the lowest level of agreement and 5 the highest level.

|  | **1** | **2** | **3** | **4** | **5** |
| --- | --- | --- | --- | --- | --- |
| The protocol should be simple and brief to integrate easily into routine primary care visits |  |  |  |  |  |
| The primary care team should receive specific training to apply the protocol consistently and respectfully |  |  |  |  |  |
| A clear procedure should exist for referral to specialized care when signs of dementia are detected |  |  |  |  |  |
| The protocol should be flexible to apply in different clinical settings, including rural consultations |  |  |  |  |  |
| The protocol should be culturally sensitive, adapting questions and tests to the language and cultural context of each community |  |  |  |  |  |

## Procedure for Individuals without an Informant

In the first round, various strategies were proposed to evaluate individuals without a reliable or available informant.

Please indicate your level of agreement that each of the following strategies should be prioritized for evaluating individuals without a reliable informant:

|  | **1** | **2** | **3** | **4** | **5** |
| --- | --- | --- | --- | --- | --- |
| Identify a valid informant with social work support, even if not a family member |  |  |  |  |  |
| Enhance collection of cognitive and functional data through medical history and clinical questions |  |  |  |  |  |
| Apply a neuropsychological assessment battery |  |  |  |  |  |
| Screening tests (e.g., MMSE, MoCA, Clock Drawing Test) |  |  |  |  |  |
| Telephone communication with a family member |  |  |  |  |  |
| Other objective tests (imaging, biomarkers) |  |  |  |  |  |
| Record a video of the patient’s behavior |  |  |  |  |  |
